# The interplay between the microbiome and colonic immune system in checkpoint inhibitor therapy

**DOI:** 10.3389/frmbi.2023.1061193

**Published:** 2023-03-03

**Authors:** Jacob Dehinsilu, Chrysi Sergaki, Gregory Amos, Vanessa Fontana, Munir Pirmohamed

**Affiliations:** ^1^ The Wolfson Centre for Personalized Medicine and Medical Research Council (MRC) Centre for Drug Safety Science, Department of Pharmacology and Therapeutics, Institute of Systems, Molecular and Integrative Biology, University of Liverpool, Liverpool, United Kingdom; ^2^ Diagnostics R&D, Medicines and Healthcare products Regulatory Agency, Potters Bar, United Kingdom

**Keywords:** checkpoint, inhibitor, PD-1, CTLA-4, gut, microbiome, colitis, IgA

## Abstract

The advent of immune checkpoint inhibitor therapy was a significant step in the development of treatments for cancer. It is, however, a double-edged sword. Immune related adverse events are the result of unleashing brakes on the immune system and affect many patients undergoing checkpoint inhibitor therapy, often being debilitating and occasionally lethal. It has been shown both in mice and in humans that the presence of certain families, genera and species of bacteria are associated with improved responses to checkpoint inhibitor therapy, whereas in their absence the response to therapy is often poor. Recent studies have demonstrated that immune related adverse events to checkpoint inhibitor therapy can be perturbed and perhaps predicted based on the composition and functional capacity of the gut microbiota and parts of the immune system. In the case of colitis associated with immune checkpoint inhibitor therapy, one interesting avenue of investigation is based on the activity of secretory immunoglobulin A (SIgA). Produced by plasma cells, IgA is present in high concentrations at the gut mucosa and is involved in both the maturation and maintenance of the microbiota as well as the development of IBD. Here we summarise the current literature surrounding the interplay between the gut microbiota and response to CPI therapy. Additionally, we overview the colonic immune system, paying particular attention to IgA, as a key component of the microbiota-immune system interaction.

## Introduction to immune checkpoint inhibitor therapy

Immune checkpoints are systems by which the adaptive immune system recognises self, thus preventing autoimmune-type reactions. When there is an immune response, immune checkpoints allow for the activity of the adaptive immune system to be altered dependent on the presentation of antigen and the presence of certain cytokines (Shi et al., 2018). One key subset of cells under the control of this system are cytotoxic T-cells. Dysregulation of these systems, favouring the inhibitory activity of immune checkpoints, can be present in tumour cells perturbing the ability of tumour specific cytotoxic T-cells to recognise and destroy cancerous cells (Pardoll, 2012).

**Figure 1:**
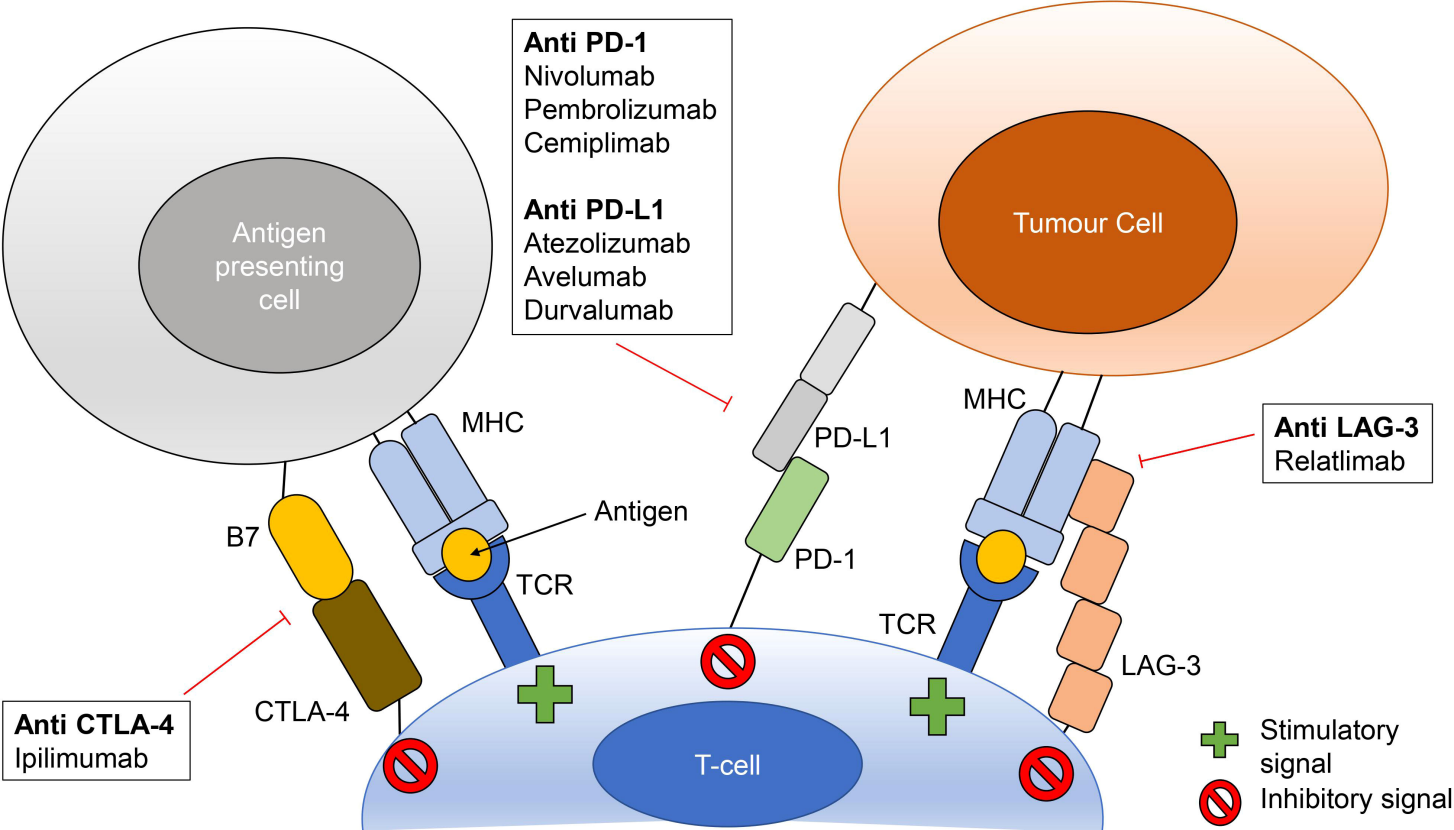
Checkpoint inhibitor therapy mechanisms of action. Checkpoint inhibitors bind to prevent interactions between CTLA-4 and B7 (B7-1 or B7-2), PD-1 and PD-L1 and LAG-3 and major histocompatibility complexes (MHC). These interactions prevent inhibitory signaling to cytotoxic T-cells thus allowing for stimulation *via* MHC-T-cell receptor (TCR) binding. Checkpoint inhibitors listed are those with FDA approval as monotherapy or as part of combination treatment.

In recent years, immunotherapy *via* checkpoint blockade has become an important tool in cancer treatment (**Figure 1**). Currently this treatment targets four cell surface immune checkpoint components: cytotoxic T-lymphocyte antigen 4 (CTLA-4), programmed cell death protein 1 (PD-1) and lymphocyte-activation gene 3 (LAG-3) on T-cells, and programmed cell death ligand 1 (PD-L1) on antigen presenting cells and cancer cells, although there are many other checkpoint molecules under investigation (Darvin et al., 2018; Tawbi et al., 2022). Checkpoint inhibitor (CPI) therapy with monoclonal antibodies specific to CTLA-4, PD-1/PD-L1 and LAG-3 help to prevent immune evasion by cancer cells by preventing these molecules from taking part in downregulation of cytotoxic T-cells, allowing tumour cells to be recognised and destroyed (Pardoll, 2012; Darvin et al., 2018; Thallinger et al., 2018).

The first example of immunotherapy using immune checkpoint inhibitors was ipilimumab (anti-CTLA-4). In terms of overall and long-term survival, this treatment demonstrated a significant improvement in patients with metastatic melanoma (Hodi et al., 2010). The development of new immune checkpoint inhibitors has allowed for both the treatment of a much larger range of cancers and the use of combination therapy. For example, in untreated melanoma it has been demonstrated that combination ipilimumab-nivolumab (anti-PD-1) therapy confers greater progression-free survival compared to ipilimumab monotherapy (Larkin et al., 2015; Postow et al., 2015b; Salem et al., 2019). The success of these drugs has massively impacted the state of research into immunomodulatory treatments for cancer over the past decade and continues to do so both in the field of new target discovery and the development of novel therapies (Xin Yu et al., 2019; Robert, 2020).

## Immune related adverse events to checkpoint inhibitor therapy

Although checkpoint inhibitor (CPI) therapy improves long term survival rates, their use can also lead to severe and life-threatening immune-related adverse events (irAE). There is a very broad spectrum of irAEs, most common being those affecting the skin, gastrointestinal tract and endocrine system (Weber et al., 2017; Das and Johnson, 2019; Spiers et al., 2019). Those contributing to the most fatalities are encephalitis, myocarditis, pneumonitis and hepatitis (Martins et al., 2019). Postow et al. demonstrated that around 90% of advanced melanoma patients receiving combination CPI therapy present with an irAE and over half of these are severe or life threatening (Postow et al., 2015b). Combination therapy has been shown to increase the likelihood of irAEs in melanoma and unfortunately, these irAEs can be a cause of treatment discontinuation (Larkin et al., 2015; Postow et al., 2015b; Postow et al., 2018).

Mechanistically, irAEs are not fully understood and it is likely that they differ depending on which CPI therapy is used (Postow et al., 2018). Postow et al. suggest that the mechanisms at play may be related to a higher amount of T-cell activity against healthy tissue, an increase in pro-inflammatory cytokines, higher levels of antecedent autoantibodies or greater complement-mediated inflammation (Callahan et al., 2011; Iwama et al., 2014; Harbour et al., 2015; Caturegli et al., 2016; Johnson et al., 2016; Byrne and Fisher, 2017; Osorio et al., 2017; Postow et al., 2018).

## Influence of the microbiome on anti PD 1/PD-L1 therapy

The human gastrointestinal tract contains a large and diverse community of bacteria, the gut microbiota (Mowat and Agace, 2014; Sender et al., 2016). The gut contains approximately 10^14^ bacteria, and each individual has a distinct set of at least 160 species from a potential repertoire of at least 1000 species (Qin et al., 2010; Li et al., 2014). The gut microbiota alone contains at least 10 times more genes than the human genome and they perform several important functions, for example, the production of B and K vitamins and the breakdown of fibrous material. They also influence several host processes including, but not limited to, immune system maturation, bile acid metabolism, hormonal regulation and neurodevelopment (Swann et al., 2011; Mulle et al., 2013; Li et al., 2014; Lynch and Pedersen, 2016; Schroeder and Bäckhed, 2016; Fan and Pedersen, 2020; He et al., 2021). The composition of microbiota is dynamic with many factors such as the environment, diet, diseases and the use of drugs such as antibiotics affecting the microbiota and its function (Kamada et al., 2013).

Due to its influence on the immune system, the role microbiota play in cancer treatment has become an important avenue of investigation (Zheng et al., 2020). Studies have already demonstrated that dysbiosis affects tumour response to platinum chemotherapy and CpG-oligonucleotide immunotherapy and that an intact microbiome is required for an anti-tumour immune response following cyclophosphamide chemotherapy (Iida et al., 2013; Viaud et al., 2013; Montassier et al., 2015).

More recently, the microbiome has also been shown to facilitate the efficacy of anti-PD 1/PD-L1 therapy, with several groups of bacteria from single species up to entire orders being significantly different in patients that respond to treatment in comparison to those that do not. Moreover, experiments in mice have demonstrated that the composition of the microbiome alone can influence tumour growth in part *via* the modulation of adaptive immunity (Sivan et al., 2015; Gopalakrishnan et al., 2018; Matson et al., 2018; Routy et al., 2018).

Sivan et al. compared melanoma growth in mice with different microbiota compositions for antitumor activity. A significant difference in tumour growth was observed and found to be the result of an immune mediated response. To observe whether this effect was lost due to a change in the microbiota, these mice were subsequently transferred to environments where their microbiome would be altered (cohousing) or were subjected to direct faecal microbiota transplant (FMT). Through this approach, coupled with 16S rRNA sequencing, *Bifidobacterium* was found to be associated with an antitumor effect. In addition, when *Bifidobacterium* was given orally, it was found to have a similar effect to anti-PD-L1 therapy. Taking this into consideration, scientists combined both *Bifidobacterium* and anti-PD-L1 therapy which had a significant effect on reducing tumour outgrowth (Sivan et al., 2015). The findings of Sivan et al. are further supported by Matson et al: stool was taken from metastatic melanoma patients before anti-PD-1 treatment, and it was found that responders had a significantly greater abundance of at least 10 different species including *Bifidobacterium adolescentis* and *Bifidobacterium longum *(Matson et al., 2018). In tandem, these studies demonstrate the potential for the microbiota, and in particular members of the *Bifidobacteriaceae* family, to be a tool that aids the prediction of treatment success, the optimisation of treatment as well as increasing the body of knowledge in support of microbiome-based therapeutics. Further investigation into how these bacteria interact with the immune system may also help us to better understand the mechanisms by which CPIs act.

The impact of the microbiota in relation to anti-PD-1/PD-L1 therapy is not determined by the composition of the microbiota alone; the level of diversity within this population also plays a role (Gopalakrishnan et al., 2018). Gopalakrishnan et al. investigated the gut and oral microbiomes in human patients undergoing anti-PD-1 treatment for melanoma to better characterise findings from mouse-based work. 16S rRNA sequencing was used to identify those bacteria associated with treatment response and progression free survival. This study demonstrated that in the gut, a high α-diversity and abundance of *Ruminococcaceae* is a major component of a more favourable microbiome. This microbiome profile correlates with individuals having a more efficient anti-tumour response and improved T-cell function (including antigen presentation to these cells) both around and within the tumour microenvironment. Contrastingly, an unfavourable microbiome which could result in an impaired anti-tumour response consisted of a low alpha diversity with a large amount of Bacteroidales present. In this case, there was a much lower infiltration of anti-tumour immune cells and a reduced amount of antigen presentation to these cells (Gopalakrishnan et al., 2018). Two studies on Chinese cohorts with non-small-cell lung carcinoma (NSCLC) undergoing anti-PD-1 treatment also found that greater microbial diversity resulted in better treatment response (Jin et al., 2019; Song et al., 2020). Given that both diversity and the presence of certain species and families of bacteria can impact response to treatment, one potential area of investigation is measurement of the degree to which they influence each other. For instance, it is important to understand the degree to which the introduction of a species common to responders alters the outcome in a non-responder with low microbial diversity.

Interestingly, Matson et al. determined that, in baseline samples *Ruminococcus obeum* is significantly more abundant in non-responders whereas in post treatment responders, Gopalakrishnan et al. found abundant *Ruminococcaceae* to be part of a favourable microbiome (Gopalakrishnan et al. report that their baseline faecal samples showed no significant change after treatment intervention) (Gopalakrishnan et al., 2018; Matson et al., 2018). One reason for this difference may be related to methodology. While both studies use 16S rRNA and shotgun sequencing, Matson et al. integrate a qPCR-based approach to define which species are enriched in responders and non-responders where Gopalakrishnan et al. do not. It is likely that this difference will impact the way in which species are classified (Gopalakrishnan et al., 2018; Matson et al., 2018).

Lee et al. investigated the gut microbiota in patients undergoing anti PD-1 therapy for unresectable hepatocellular carcinoma. They found that objective response was associated with the presence of *Lachnoclostridium*, *Lachnospiraceae*, and *Veillonella*. The abundance of *Lachnoclostridium* was also correlated with the presence of ursodeoxycholic acid and ursocholic acid both of which were associated with an objective response (Lee et al., 2022). Further study into these species and bile acids may reveal mechanisms by which the gut microbiota specifically influence anti PD-1 therapy responses in hepatocellular carcinoma. The interaction between the gut microbiota and the liver is multifaceted. The gut microbiota aid in the regulation of bile acid synthesis and glucose/lipid metabolism whilst bile acids produced in the liver aid in preventing aberrant bacterial outgrowth (Tripathi et al., 2018; van Treuren and Dodd, 2020). Conversely, disruption of the gut and dysbiosis has been associated with the development of chronic liver disease and hepatocellular carcinoma (Zheng and Wang, 2021). When assessing the way in which the gut microbiota and CPI-therapy are linked, it is important to consider the global reach of the gut microbial products and immune system induction. Appreciation of these interactions may help us better understand the underlying mechanisms by which gut microbiota impacts CPI-therapy response in different tissues.

Two recent phase 1 clinical trials investigated the use of FMT in patients undergoing anti PD-1 therapy for melanoma (Baruch et al., 2021; Davar et al., 2021). Baruch et al. transplanted stool from one of two donors who had complete response to anti PD-1 therapy into a set of 10 patients. Antibiotic treatment was used to deplete the native microbiome followed by colonoscopy and administration of capsulised FMT (orally). A maintenance period began consisting of a further 12 days of capsulised FMT followed by anti PD-1 treatment on day 14. This cycle was repeated 6 times with anti PD-1 monotherapy continuing after the combination treatment. Of the 10 patients a clinical response was observed in 3 patients with one having a complete response. Baruch et al. found that treatment was associated with favorable changes in immune cell infiltration and gene expression in both the tumor microenvironment and gut lamina propria (Baruch et al., 2021).

Davar et al. transplanted stool endoscopically from one of 7 patients with a partial or complete response to CPI therapy to 15 melanoma patients who showed no response to prior CPI therapy and had progressive disease; this was followed by anti PD-1 therapy every three weeks. Of the 15 patients, 3 had an objective response to treatment and 3 others had stable disease for >12 months. The composition of the fecal microbiota was shown to impact the metabolome and cytokine profile of responders as well as regulate distinct biological signatures seen in this patient group. Responders also showed increased activation of CD8+ T cells and a decrease in the frequency of interleukin-8 (IL-8) expressing myeloid cells (higher serum IL-8 is associated with a reduction in the clinical benefit of CPI therapy) (Schalper et al., 2020; Davar et al., 2021).

These studies demonstrate the safety, viability, and potential therapeutic benefits of a combination anti PD-1-FMT treatment. Studies with greater statistical power are needed to optimize donor selection, the procedure for FMT itself (for example the use of antibiotics and method/frequency of transplantation) and which pre-treatment patient phenotypes are associated with an objective response.

In summary, the complexity of the microbiome and how it might influence the immune system in the setting of anti-PD 1/PD-L1 therapy presents a mammoth task. Further studies are vital to determine not only how bacteria identified as promoting treatment success act mechanistically but also how bacteria-bacteria interactions may play a role. This will aid in current searches for effective microbiota-based treatments to supplement CPI therapy.

## Influence of the microbiota on anti-CTLA-4 therapy

Like anti-PD 1/PD-L1 therapy, gut microbiota can also affect anti-CTLA-4 therapy. While this is less well studied in the context of the microbiome (and less frequently used alone clinically) than anti PD 1/PD-L1 therapy, studies on the augmentation of anti-CTLA-4 therapy by the microbiome remain important (Postow et al., 2015a; Dubin et al., 2016; Shi et al., 2018).

Vetizou et al. were able to show a significant role of the microbiome for successful anti-CTLA-4 treatment using germ-free mice. They found that germ free mice were unresponsive to anti-CTLA-4 treatment, whereas with the introduction of *Bacteroides fragilis* cells, cellular components and T-cells matured in response to the presence of bacteria, and the effect of CTLA-4 blockade was restored (Vétizou et al., 2015). This study demonstrates that the microbiome impacts anti-CTLA-4 as well as anti-PD-1/PD-L1 therapies. It also provides evidence that there may be potential, in the future, for research into microbiome-based therapies based on individual cellular components or specially primed cells. Currently, microbiota-based therapies to supplement CPI therapy are in early clinical trials, based on both individual bacteria and communities consisting of multiple species (Zipkin, 2021).

The impact of the microbiome in anti-CTLA-4 therapy is as important as in anti-PD-1 therapy as they are often used in combination (Postow et al., 2015b). In addition, phase 3 trials using this combination to treat NSCLC also show promise and there are ongoing clinical trials for new anti-CTLA-4 therapies (Hellmann et al., 2019; Sharma et al., 2020). Further research into *Bacteroidales* being part of a favourable microbiome in anti-CTLA-4 therapy but unfavourable in anti PD-1 therapy may also help to elucidate the mechanisms by which bacteria influence the immune system to impact the outcome of CPI therapy (Vétizou et al., 2015; Gopalakrishnan et al., 2018).

## The role of the Microbiota in irAE colitis

IrAE colitis after the treatment with checkpoint inhibitors is mediated by a range of factors including genetics, autoimmunity, the gut microbiota and the environment (Som et al., 2019; Baradaran Ghavami et al., 2021; Chennamadhavuni et al., 2022). It is characterised by over-stimulation of T-cell mediated immunity to certain enteric bacteria and is most often triggered by an environmental factor interfering with gut homeostasis such as a break in the mucosal barrier or some form of dysbiosis (Sartor, 2006; Kadijani et al., 2018). It is among the most common irAEs when it comes to both anti-CTLA-4 treatment and combination anti-CTLA-4/PD-1 therapy. However, it should be noted that its fatality rate is much lower (2-5%) compared to myocarditis (39.7%), for example. (Wang et al., 2018a)

Certain members of the intestinal microbiota have been shown to both predict and improve clinical outcomes with regards to colitis resulting from ipilimumab treatment for melanoma. Wang et al. demonstrated this in a mouse melanoma model (Wang et al., 2018b). Following treatment with anti-CTLA-4, dextran sodium sulphate was then introduced to induce colitis in these mice. They found that susceptibility to colitis, weight loss and histologic scores were greater in mice receiving anti-CTLA-4 treatment compared to those receiving an isotype control. The colitis phenotype was then reduced in severity upon introduction of *Bifidobacterium* (Wang et al., 2018b; Baradaran Ghavami et al., 2021). If similar mechanisms to these occur in humans, then *Bifidobacterium* may represent a cheap way to improve patient care by reducing the severity of irAE colitis.

Dubin et al. used metagenomic shotgun sequencing to both identify bacteria present in the microbiome that are associated with resistance to colitis following ipilimumab treatment and identify metabolic pathways that may be involved. In pre-treatment faecal samples, increased representation of the Bacteroidetes phylum (including members of the Bacteriodaceae family) correlated with resistance to ipilimumab induced colitis in metastatic melanoma patients (Dubin et al., 2016). In addition, they used shotgun metagenomic sequencing coupled with computational analysis to determine the functional capability of the microbiotas of patients that remained colitis free and those that progressed to colitis following treatment (Segata et al., 2011; Kanehisa et al., 2021). They demonstrated that protection against irAE colitis following ipilimumab treatment was associated with bacteria able to take part in the production of B vitamins (B1, B2 and B5) and polyamine transport (Dubin et al., 2016). Levels of vitamins B1 and B2 are reduced in Crohn’s disease, and they have been linked to B-cell differentiation due to their role in metabolism (Yoshii et al., 2019). Vitamin B2 plays an anti-inflammatory and immune modulatory role in the gut (Kuroki et al., 1993; Yoshii et al., 2019; Von Martels et al., 2020). Vitamin B5 deficiency promotes inflammation(Berruyer et al., 2006; Zhang et al., 2011; Yoshii et al., 2019). Polyamines have been linked to several processes but are important in cellular turnover and differentiation of the intestinal mucosa (McCormack and Johnson, 1991). Studies also demonstrate that processing of the polyamine spermine by spermine oxidase (SMOX) plays an active role in inflammation. In mice the activity of SMOX is protective against DSS induced colitis whilst not being protective against colitis and gastric inflammation caused by *Citrobacter rodentium* and *Helicobacter pylori*, respectively. The method of analysis in this study gives insight not only into which bacteria are associated with resistance to colitis but also how they might interact with the immune system. Moreover, functional characterisation of microbiota associated with irAE resistance may elucidate biomarkers that can be used to predict the likelihood of irAEs.

Chatput et al. grouped metastatic melanoma patients undergoing anti CTLA-4 treatment based on their microbiota composition. Those patients whose baseline microbiota contained more Firmicutes (in particular *Faecalibacterium*) had both greater survival than those whose baseline microbiota was more dominated by *Bacteroides*. Additionally, a higher frequency of treatment induced colitis was associated with Firmicutes (Chaput et al., 2017). As in Dubin et al, the presence of *Bacteroides* correlated with reduced colitis. This demonstrated that the effect of the gut microbiome is not just limited to treatment effectiveness, but it can also impact the safety of a treatment with regards to irAEs and therefore the likelihood of treatment success.

Our understanding of the relationship between irAE colitis and the microbiota may be enhanced by considering the relationship between microbiota and inflammatory bowel disease (IBD). IBD mainly comprises Crohn’s disease and ulcerative colitis, both of which are the result of aberrant inflammatory responses, the cause of which remains elusive (Sartor, 2006; Khan et al., 2019). Both conditions have similarities with some overlapping symptoms and there are several genes that play a role in susceptibility to one or both conditions. These disorders also differ in terms of localisation. Normally, ulcerative colitis is continuous and restricted to the colonic mucosa whereas Crohn’s can affect any part of the intestinal tract from mouth to anus, and can be discontinuous with inflammation often being transmural (Abraham and Cho, 2009; Gajendran et al., 2019; Sartor, 2006).

Various changes in the gut microbiota have been associated with IBD with reduced overall diversity, increases in proteobacteria and decreases in Firmicutes being the most consistent finings in human studies (Khan et al., 2019). While mouse models demonstrate that certain species can stimulate or dampen the host inflammatory response, no bacterium or group of bacteria have been conclusively shown to have a causative relationship with IBD. Indeed, whether dysbiosis is the causative agent or a consequence of IBD remains a contentious topic (Nell et al., 2010; Gkouskou et al., 2014; Matsuoka and Kanai, 2015).

*Faecalibacterium prausnitzzi* is often found to be decreased in IBD patients. Interestingly however, Chaput et al. show an association between *F. prausnitzzi* (and other Firmicutes) and an increase in the occurrence of irAE colitis following anti-CTLA-4 treatment (Chaput et al., 2017; Khan et al., 2019). Examining the differences in *F. prausnitzzi* activity in non-pharmacological IBD and irAE colitis may help to elucidate pathways by which bacteria influence the immune system and drive or prevent inflammation in the gut.

## The impact of antibiotic treatment on immune checkpoint inhibitor therapy outcomes

Patients undergoing treatment for cancer may have compromised immune systems as the result of disease or immune perturbation due to treatment. Antibiotic intervention may therefore be necessary to fight bacterial infections (Gao et al., 2020). While antibiotic treatments reduce the risk of morbidity due to infection, they may also have significant negative impacts on mutualism between a patient and their gut microbiota (Ramirez et al., 2020). Multiple studies have linked antibiotic exposure to a reduction in treatment response, overall survival and progression-free survival (see Pinato et al. for a summary of such studies) (Pinato et al., 2019). Antibiotic treatment has also been shown to impact the development of irAEs to CPI therapy. Abu-Sbeih et al. found that in patients undergoing CPI therapy, antibiotic exposure was associated with decreased immune mediated diarrhea/colitis. However, administration of antibiotics with anerobic activity was associated with a higher risk of severe immune mediated diarrhea/colitis and the likelihood of this was greater when antibiotics were given after administration of CPI therapy (Abu-Sbeih et al., 2019).

The use of antibiotic therapy adds further complications to research surrounding the CPI therapy-microbiota axis. It will be important to assess how antibiotic usage (both pre and post CPI-therapy) effects the composition of the gut microbiota and to what degree it’s disruption impacts patient outcomes, the development of adverse events and how this occurs mechanistically.

## The role of B-cells in microbiota-immune checkpoint inhibitor therapy interactions

The role of B cells in cancer is much less well characterised than that of T cells. B cells take part in anti-tumour immunity *via* antigen presentation to T-cells, proinflammatory cytokine production and complement activation and in a pro-tumour role by downregulating T cell mediated immunity (Largeot et al., 2019). In the context of CPI therapy, this gulf in characterisation remains (see Malczewski et al. for a summary of how SCFAs impact T-cell differentiation) (Malczewski et al., 2021).

Recent evidence points to an active role for B-cells in both the efficacy of CPI therapy as well as the resultant irAEs. A greater proportion of CD20+ B-cells combined with greater tertiary lymphoid tissue area relative to the tumour, increases in plasmablasts, switched memory B-cells and certain IgG subtypes all correlate with a favourable response to CPI therapy (Diem et al., 2019; Fässler et al., 2019; Griss et al., 2019; Helmink et al., 2020; Willsmore et al., 2020). Following combination CPI therapy in advanced melanoma patients, Das et al. found that there was a decrease in peripheral blood B cells but an increase in CD21lo B cells and plasmablasts. In addition, those patients with early B cell changes were more likely to develop an irAE following combination therapy (Das et al., 2018). Recent evidence suggests that CD21lo cells are a subset primed to differentiate into plasma cells (Lau et al., 2017). This demonstrates that B cells and their subpopulations may be a useful indicator of irAE development following combination CPI therapy. While it is not clear how these changes are related to irAEs specifically, B cells are implicated in several autoimmune conditions where they have been shown to produce autoantibodies, secrete pro inflammatory cytokines and promote the generation of ectopic lymphoid tissue (Marston et al., 2010; Hampe et al., 2012; Palaiologou et al., 2014). The correlation between combination checkpoint inhibitor therapy and an increase in CD21lo and plasmablasts may also indicate a role for antibody production. These mechanisms may provide potential avenues of investigation into the role of B cells in CPI therapy induced irAEs.

## The role of immunoglobulin A in the colon

A healthy colon will contain microorganisms in a mutually beneficial relationship with the host and it is vital that this balance is maintained. Indeed, this section of the gastrointestinal tract contains orders of magnitude more bacteria per ml than the small intestine (up to 10^5^/ml in the jejunum versus up to 10^12^/ml in the caecum and colon) making aberrant microbial activity particularly dangerous. For homeostasis to be maintained, the immune system employs a multifaceted approach in both surveillance and response.

The colonic mucosa is exposed to a myriad of agents, both biological and from digestion, presenting a challenge to gut homeostasis. Immunoglobulin A (IgA) is a key player in its maintenance and protection (Pabst et al., 2016). In the gut lumen, IgA exists as secretory IgA (SIgA). B cells mature into IgA producing plasma cells either in a T-cell dependent (Td) or independent (Ti) manner. The Td pathway takes place in organised lymphoid tissue. This pathway relies on antigen binding at the B cell receptor, presentation of antigen to a T helper cell, interactions between CD40 and its ligand CD40L and the action of several cytokines. The Ti pathway occurs in the lamina propria and does not rely on presentation of antigen to T-cells, but rather the B-cells are activated by B-cell receptor crosslinking or *via* toll like receptors (Gutzeit et al., 2014). In addition, the antigens tend to be simple and repetitive in the case of the Ti pathway (e.g., a repetitive carbohydrate). Most gut bound IgA is secreted by plasma cells residing in the organised lymphoid tissue as polymeric IgA (pIgA). Following transcytosis through the gut epithelium, binding to and cleavage of its receptor (that forms the secretory component), PIgA becomes SIgA (Pabst, 2012; Gutzeit et al., 2014).

Immunoglobulin binding is typically thought of as an interaction between the complementarity determining region of a Fab arm and a specific antigen because of somatic hypermutation, called canonical binding. However, SIgA can also take part in binding independent of the canonical frame (Pabst and Slack, 2020). One key component of this non-canonical binding is the heavy glycosylation of SIgA comprising O-glycan attachment at hinge regions and N-glycan attachment on both the secretory component and J chain. It has also been proposed that this glycosylation may play a competitive inhibitory role because of similarities to the glycosylation on the epithelium exposed to the gut lumen (Royle et al., 2003; Mantis et al., 2011). It is important to note that canonical binding of a particular SIgA clone is not restricted to a single species of bacteria allowing for cross-species reactivity (given enough epitope similarity). Also, a combination of canonical and non-canonical SIgA binding by the same clone can also facilitate cross species reactivity (Bunker et al., 2017; Pabst and Slack, 2020).

**Figure 2:**
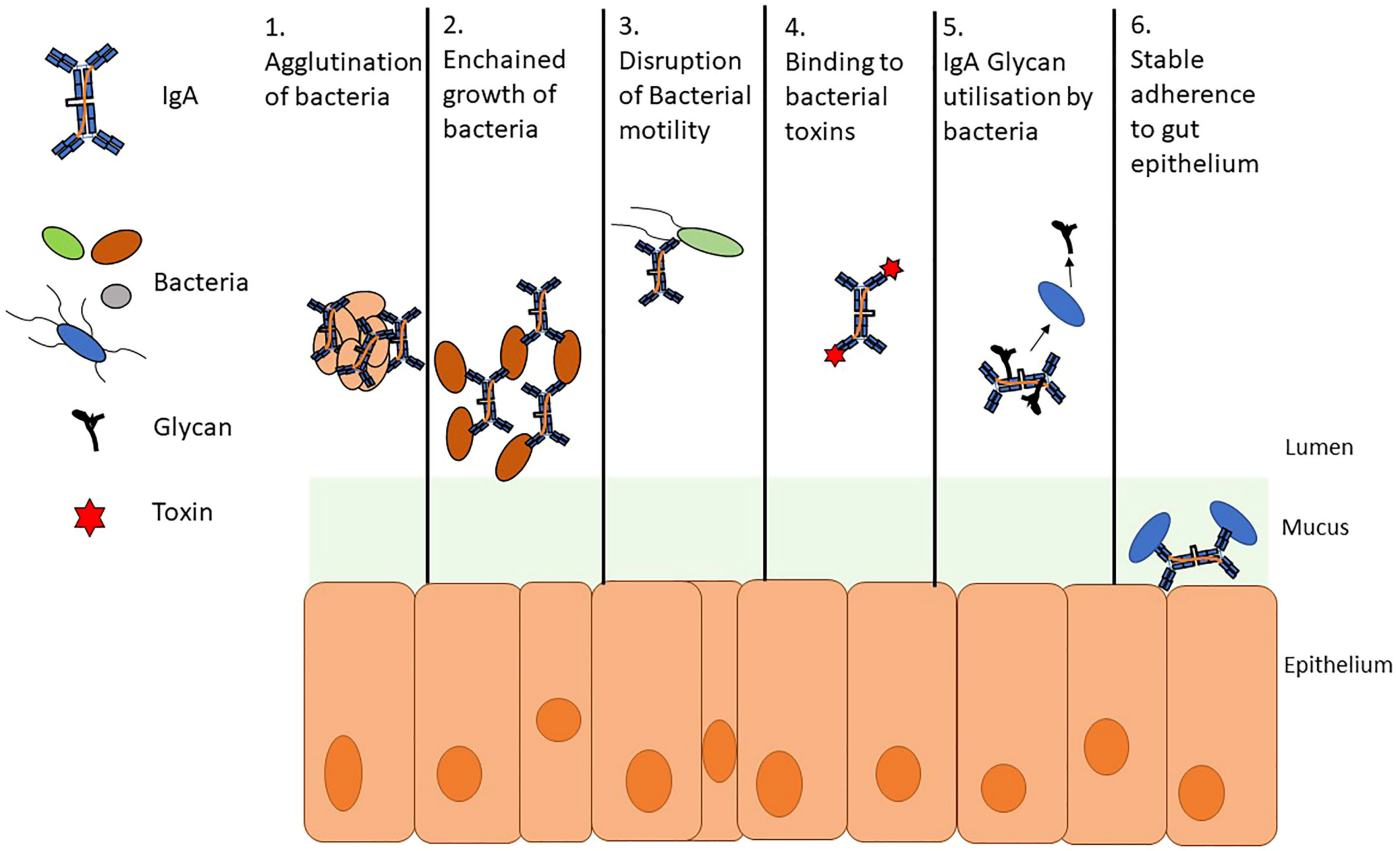
A summary of secretory SIgA activity. SIgA takes part in several interactions with members of the gut microbiome. These interactions both impede and promote colonization of bacteria. 1. Agglutination and 2. Enchained growth are similar and potentially complementary processes; however, enchained growth requires a lower cellular density than agglutination to clump bacteria for clearance. 3. Bacterial motility can be disrupted by agglutination and enchained growth but also by binding to flagella and immunoglobulin mediated downregulation of the genes involved in flagellar motility. 4. SIgA has been shown to hinder bacterial pathogenesis by directly binding to toxins and by preventing interactions with the gut epithelium 5. Glycans can be utilized by members of the microbiota in several metabolic processes. Bacteroides spp. can cleave glycans from SIgA 6. Bacteria can trigger a specific IgA response allowing for stable adherence to gut epithelium.

The role of SIgA is multifaceted but remains incompletely understood (**Figure 2**) (Mantis et al., 2011). It is often described as a first line of defense at the mucosa making up part of the physical barrier against pathogens and antigens, protecting the gut epithelium and its surface structures; this role is known as immune exclusion (Stokes et al., 1975). SIgA, for example, can physically bind to cholera toxin. This interaction prevents the toxin from interacting with monosialotetrahexosylganglioside at the gut epithelium thus perturbing its toxic effect (Mantis et al., 2011). SIgA also binds to common surface antigens and allows for the agglutination (clumping together of cells and particles) and enchained growth of pathogenic bacteria *via* crosslinking which may aid clearance (Pabst and Slack, 2020). In the case of *Salmonella enterica* serovar Typhimurium, a monoclonal IgA known as Sal4 (derived from IgA producing hybridoma cells) can form crosslinks between O-antigen epitope disrupting motility *in vitro* and limiting the translocation of effector proteins in mice *via* outer membrane distortion (Michetrti et al., 1992; Mantis et al., 2011; Richards et al., 2020). Furthermore, there is evidence to suggest that immunoglobulin binding can negatively impact motility and downregulate the expression of flagellar genes (Cullender et al., 2013). SIgA also has the ability to promote colonization in the gut and may play roles in microbial metabolism (Koropatkin et al., 2012; Donaldson et al., 2018; Briliūtė et al., 2019).

Further important information on this subject comes from studies of IgA-deficiency. Although the phenotype of IgA-deficiency is described as asymptomatic for the majority of those with the condition, some individuals are more likely to develop gastrointestinal disorders, auto immune diseases and lung infections (Domínguez et al., 2012; Yazdani et al., 2017). Additionally, there is an effect on the microbiome composition, namely a decrease in microbial diversity and increase in potentially proinflammatory microbiota members (Yazdani et al., 2017; Moll et al., 2021). In *Aid* ^-/-^ mice (*Aid* is required for class switch recombination and somatic hypermutation), there is expansion of anaerobic bacteria, notably segmented filamentous bacteria (SFB)(Suzuki et al., 2004). In healthy mice, SFB are implicated in maturation of the immune system *via* stimulation of Th17 mediated immunity and the production of IgA involved in gut homeostasis, although the *Aid* ^-/-^ phenotype prevents this (Lécuyer et al., 2014; Flannigan and Denning, 2018). Anastomosis of *Aid* ^-/-^ mice to mice with a normal immune system, allows for the transfer of gut B-cells which produce hypermutated IgA. The presence of this IgA results in a microbiota more closely resembling wild type mice (Suzuki et al., 2004; Ivanov et al., 2008; Ivanov and Littman, 2010).

In another study, Palm et al. demonstrated that bacteria highly coated in IgA represent a colitogenic population. The bacteria that were found to be most highly coated in IgA in this instance (*Prevotellaceae*, *Helicobacter*, and SFBs) are known key players in colitis pathogenesis. Furthermore, colonisation with highly coated bacteria identified and cultured from IBD patients resulted in a higher likelihood of colitis in a germ-free mouse model (Palm et al., 2014).

## Influence of microbial metabolism on the colonic immune system

In many cases the relationship between the microbiota and host is mutually beneficial; a key example of this is the digestion of fibrous material to produce short chain fatty acids (SCFA) in the gut (Scheppach, 1994; Venegas et al., 2019). These comprise carboxylic acids with fewer than 6 carbons, the most common of which are acetate (2 C), propionate (3 C) and butyrate (4 C) which, in the colon, are found in a molar ratio of 3:1:1 (Cummings et al., 1987). Acetate is a key growth factor for some microbes as well as an important metabolite in host production of cholesterol and lipids. Propionate has a less well-defined role in the gut but plays an important role in the liver (Hosseini et al., 2011). Butyrate is an energy source for colonocytes (via β-oxidation to acetyl-CoA) and has been shown to play a role in helping to maintain the integrity of the gut epithelium *via* upregulation of Claudin-1 (a tight junction protein) *in vitro* (Clausen and Mortensen, 1995; Wang et al., 2012; Morrison and Preston, 2016).

Fecal SCFA concentrations may also have links to CPI therapy response. Literature on this topic is limited; however, there is evidence to suggest that the presence of butyrate has a negative impact on the efficacy of anti-CTLA-4 treatment whereas greater concentrations of SCFAs are associated with improved progression free-survival in patients treated with anti PD-1 therapy (Coutzac et al., 2020; Nomura et al., 2020). B-cells are a commonality between IgA production and microbial metabolism and their activity may offer a potential avenue of investigation into how microbes impact the immune system during checkpoint inhibitor therapy.

SCFAs promote the expression of genes needed for B-cell differentiation into plasma cells (Kim et al., 2016). Kim et al. demonstrated that the SCFAs produced by the microbiota increase acetyl-CoA levels in B-cells while promoting the metabolic pathways needed to supply the raw materials for high levels of antibody production (Kim et al., 2016). In addition, SCFAs were found to promote the *in vitro* expression of 5 key genes:

1. *AICDA*, a cytidine deaminase gene involved in somatic hypermutation and class switching (Xu et al., 2007).
2. *PDRM1* (*BLIMP1*) which has a role in the differentiation of antibody producing cells (Tellier et al., 2016)
3. *SDC1* (CD138) which is a marker of mature plasma cells (McCarron et al., 2017).
4. *IRF4*, a transcription factor which regulates initial expression of *PDRM1* that is also essential for survival in B-cells due to mitochondria regulation (Low et al., 2019)
5. *XBP1*, an essential regulator of *IRF4* and *PDRM1* (Cocco et al., 2012; Kim et al., 2016).

One way in which SCFAs can alter cell activity is by interacting with G-protein coupled receptors GPR41 and GPR43 (SCFAs can also act by altering epigenetic markers *via* histone deacetylase activity) (Brown et al., 2003; Wu et al., 2017). Wu et al. demonstrated that acetate binding to GPR43 promotes an intestinal IgA response. An increase in acetate supplementation increases the expression of IgA in WT mice while the GPR43^-/-^ genotype results in reduced expression of IgA that is not rescued by acetate supplementation. They found that this is a result of vitamin A conversion to retinoic acid by dendritic cells, which has been shown to facilitate IgA class switching (Seo et al., 2014; Marks et al., 2016; Wu et al., 2017).

Recent evidence points to an active role for B-cells in both the efficacy of CPI therapy as well as resultant irAEs. A greater proportion of CD20+ B-cells combined with greater tertiary lymphoid tissue area relative to the tumour, increases in plasmablasts, switched memory B-cells and certain IgG subtypes all correlate with a favourable response to CPI therapy (Diem et al., 2019; Fässler et al., 2019; Griss et al., 2019; Helmink et al., 2020; Willsmore et al., 2020). Following combination CPI therapy in advanced melanoma patients, Das et al. found that there was a decrease in peripheral blood B cells but an increase in CD21lo B cells and Plasmablasts. In addition, those patients with early B cell changes were more likely to develop an irAE following combination therapy (Das et al., 2018). Recent evidence suggests that CD21lo cells are a subset primed to differentiate into plasma cells (Lau et al., 2017). This demonstrates that B cells and their subpopulations may be a useful indicator of irAE development following combination CPI therapy. While it is not clear how these changes are related to irAEs specifically, B cells are implicated in several autoimmune conditions where they have been shown to produce autoantibodies, secrete pro inflammatory cytokines and promote the generation of ectopic lymphoid tissue (Marston et al., 2010; Hampe et al., 2012; Palaiologou et al., 2014). The correlation between combination checkpoint inhibitor therapy and an increase in CD21lo and Plasmablasts may also indicate a role for antibody production. These mechanisms may provide potential avenues of investigation into the role of B cells in CPI therapy induced irAEs.

## Conclusion

The impact of CPI therapy on patient outcomes has been significant, providing increases in both overall and long-term survival for eligible patients. The broader oncology landscape will also be impacted by the introduction of new checkpoint inhibitor molecules targeting lymphocyte activation gene-3 (LAG-3) and T cell immunoglobulin and mucin-domain containing-3 (TIM-3) expanding the subset of patients for which checkpoint blockade is viable and allowing for new combination treatments(Qin et al., 2019).

Many questions about the link between the microbiome-checkpoint inhibitor therapy interaction remain, for example:

1. What role, if any, will gut microbiome composition and diversity play in the recently approved combination nivolumab (anti-PD1)-relatlimab (anti-LAG-3) therapy?
2. Are there specific features of a bacterium that result in it being found more frequently or in greater abundance in responders vs non-responders to CPI therapy?
3. To what degree does the level of standardisation between labs impact the results of research into the gut microbiome-CPI therapy interaction.
4. Does the relationship between IgA coating of bacteria and non-pharmacological colitis change in the context of irAE colitis?

There is strong evidence to suggest that the presence of several components of the gut microbiota are associated with response to CPI therapy, while others are associated with resistance to CPI induced colitis (Sivan et al., 2015; Vétizou et al., 2015; Dubin et al., 2016; Chaput et al, 2017; Gopalakrishnan et al., 2018; Matson et al., 2018; Wang et al., 2018b; Peng et al., 2020; McCulloch et al., 2022). These studies have already given rise to CPI therapy-microbe combination clinical trials and may yet be a foundation for more optimal personalised treatments based on a patient’s own microbiota composition (Zipkin, 2021).

## Author contributions

JD wrote the first draft of the manuscript. JD discussed this publication and received feedback from CS, GA, VF and MP. JD revised the publication with input and comments from CS, GA, VF and MP. All authors contributed to the article and approved the submitted version.
